# Effects of Barley (*Hordeum vulgare* L.) on Antioxidant Activities of Maillard Reaction Products and Consumer Acceptance of Barley Nurungji, Traditional Korean Snack

**DOI:** 10.3390/foods14040655

**Published:** 2025-02-15

**Authors:** Yerim Jeong, Il Sook Choi

**Affiliations:** 1 Department of Food and Nutrition, Wonkwang University, Iksandae-ro, Iksan 54538, Republic of Korea; oiirimee@naver.com; 2 Institute for Better Living, Wonkwang University, Iksandae-ro, Iksan 54538, Republic of Korea

**Keywords:** nurungji, waxy barley cultivar, electric pressure rice cooker, *β*-glucan, Maillard reaction products, antioxidant activity, consumer acceptance

## Abstract

This study evaluated the physicochemical characteristics, antioxidant properties, and consumer acceptance of a traditional Korean snack nurungji blended with barley (*Hordeum vulgare* L.). The antioxidant activity of *β*-glucan was identified in barley using a model Maillard reaction products (MRPs) system. Nurungji samples were analyzed based on barley cultivars (waxy and hulled), barley blending ratios (0, 25, 50, 75, and 100), and cooking equipment (electric and electric pressure rice cooker). Nurungji blended with waxy barley and cooked using an electric pressure rice cooker showed a significant increase in antioxidant properties, such as 2,2-diphenyl-1-picrylhydrazyl and 2,2′-azinobis-(3-ethylbenzothiazoline-6-sulfonic acid) radical scavenging activities, ferric reducing antioxidant power, and reducing power. The antioxidant activity of the model MRP solutions increased according to heating time and the addition of *β*-glucan. In the consumer acceptance test, nurungji blended with 50% barley showed a significantly higher acceptance rate in terms of overall evaluation, aroma, taste, texture, aftertaste, and purchase intents.

## 1. Introduction

Rice (*Oryza sativa* L.) is a significant grain consumed by over half the world’s population. It is a major source of calories, containing approximately 80% carbohydrates of grain dry weight, 7% proteins high in glutamic and aspartic acids with limiting amino acids (lysine), and 0.4% lipids with various essential vitamins and minerals [1]. Globally, rice production has increased by 1% annually, from 500 million tons in 2014 to 522.62 million tons in 2024 [2]. Despite this international trend, rice production in South Korea has declined, driven by the rise of nuclear families and single-person households [3]. Rice production decreased from 4230 thousand tons in 2013 to 3702 thousand tons in 2023, with rice consumption declining from 65.1 kg/person in 2014 to 56.4 kg/person in 2023 [4]. However, consumption of processed rice products, such as rice noodles, rice cakes, rice paper, gluten-free rice products, and nurungji, has increased in Republic of Korea [5].

Nurungji is a traditional Korean snack that develops when a thin crust forms on the bottom of a pot as the moisture evaporates from cooked rice [6]. When the temperature of the bottom side of the pot exceeds 200 °C, nurungji develops a savory flavor through a Maillard browning reaction [7]. The snack is often consumed as a drink or soup, eaten as dessert or breakfast, or used as an ingredient in recipes [8,9,10]. The versatility of nurungji has led to increased consumer demand and interest in nurungji as a gluten-free product and flour substitute [3]. Studies have been reported to improve quality properties of physicochemical, antioxidants, and sensory characteristics by adding ingredients such as turmeric powder [11], green whole grain [12], different types of rice cultivars [13], different milling degrees of rice [8], and *Dioscorea japonica* powder [14]. Additionally, nurungji extraction methods for flavor [9], quality evaluation of commercial nurungji [7], and nurungji cooking conditions and processing methods have been studied extensively [10,15,16,17,18].

Barley (*Hordeum vulgare* L.) is one of the most widely cultivated grains after rice, wheat, and corn. Barley is primarily used for brewing, malting, and feeding animals [19]. In addition, barley is a functional food ingredient containing polyphenols and carbohydrates, constituting approximately 78–83% of the total dry weight, including non-structural polysaccharides and non-starch polysaccharides. The grain contains 7–25% protein, 3–3.5% lipids, vitamins, and minerals [19,20]. The functional ingredients of barley, such as phenolic compounds and *β*-glucan, are linked to health benefits, including improved intestinal function and reduced serum cholesterol and blood glucose [20]. These health benefits have increased the use of barley in the food industry [21,22]. Various properties of barley, such as pigmented barley and barley sprout, have been studied to enhance its applicability [23,24]. Barley can be classified into hulled and hull-less barley based on the presence of the outer husk, and waxy and non-waxy barley based on the ratio of amylose to amylopectin. Hulled barley is mainly malted, whereas hull-less barley is commonly cooked. Moreover, *β*-glucan levels in hull-less barley are higher than those in hulled barley [19,20]. Considering that barley contains *β*-glucan, nurungji blended with barley may inherit physicochemical and antioxidant characteristics from barley based on barley varieties and barley blending ratios. Therefore, understanding the quality characteristics of nurungji blended with barley (hulled and waxy) could provide valuable information for those seeking healthy gluten-free snacks.

Thus, this study hypothesized that waxy barley, which contains more starch than that in hulled barley, would improve the antioxidant properties of nurungji via the Maillard reaction. Additionally, an electric pressure rice cooker would be more effective in enhancing the antioxidant properties of nurungji [17]. This study further hypothesized that *β*-glucan, a functional component of barley, would affect the antioxidant properties of nurungji. Lastly, this study hypothesized that consumer acceptance of nurungji blended with barley would be higher than that of nurungji without barley in terms of sensory acceptance (color, taste, texture, and aftertaste).

Hence, this study focused on identifying the physicochemical and antioxidant properties of nurungji made with two types of barley varieties and two types of cooking equipment, with the aim of applying these findings in the snack industry for rice-based products. Furthermore, this study aimed to clarify the effect of *β*-glucan on the Maillard reaction products (MRPs) model system to identify whether *β*-glucan from barley affects the antioxidant properties of nurungji. Finally, this study evaluated the consumer acceptance of nurungji blended with barley under various barley blending conditions to identify the sensory attributes that drive consumer preference.

## 2. Materials and Methods

### 2.1. Materials

Rice (*Oryza sativa* L.), waxy barley, and hulled barley for nurungji preparation were purchased from Iksan, Republic of Korea. The grains were then stored in a refrigerator (C110AK, LG Electronics, Seoul, Republic of Korea). L-lysine, D-(+)-glucose, D(−)-fructose, and *β*-glucan, which were used to identify the antioxidant activity of *β*-glucan in barley using model MRP solutions, were obtained from Sigma-Aldrich (Schnelldorf, Germany) and Megazyme Ltd., Bray (Wicklow County, Ireland). The 2,2-diphenyl-1-picrylhydrazyl (DPPH), Folin–Ciocalteu’s reagent, ferric chloride hexahydrate, 2,2′-azino-bis (3-ethylbenzothiazoline-6-sulfonic acid) (ABTS), 2,4,6-tri(2-pyridyl)-s-triazine, and Trolox, which were used to clarify the antioxidant activity of nurungji blended with barley, were obtained from Sigma-Aldrich.

### 2.2. Preparation of Barley Nurungji

**Figure 1:**
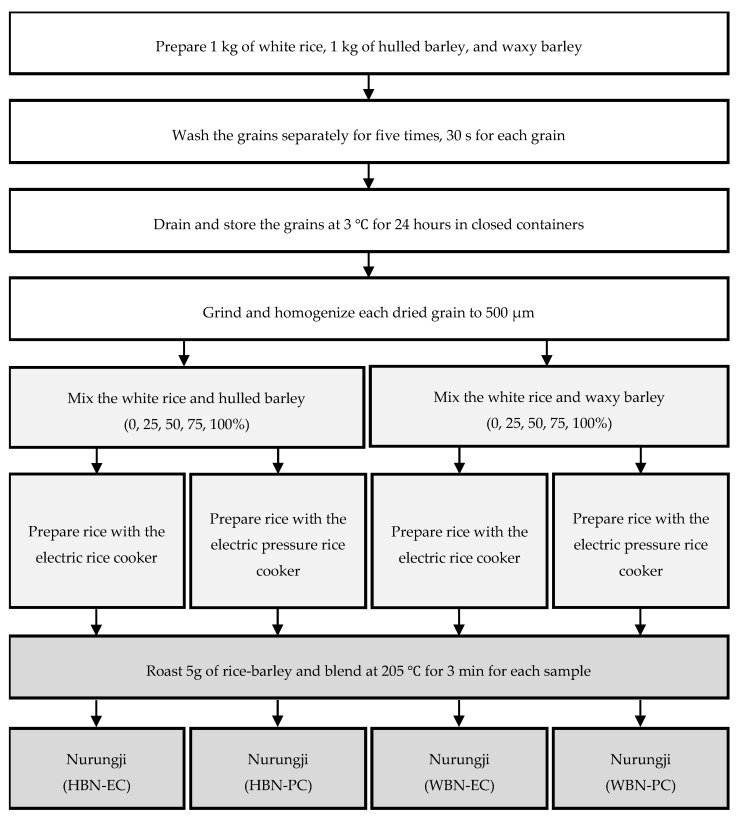
Preparation of nurungji fortified with barley (*Hordeum vulgare* L.) by using the different types of cooking equipment, barley varieties, and barley addition ratios. Hulled barley nurungji using an electric rice cooker (HBN-EC), hulled barley nurungji using an electric pressure rice cooker (HBN-PC), waxy barley nurungji using an electric rice cooker (WBN-EC), waxy barley nurungji using an electric pressure rice cooker (WBN-PC).

The barley nurungji preparation process consisted of three steps: preprocessing, cooking, and preparation (Figure 1). In the preprocessing step, 1 kg of rice and barley were each washed five times for 30 sec with water (1:2 grain-to-water ratio). During the third wash, water was drained, and the grains were rubbed clean. After washing, the grains were drained and stored in a refrigerator to dry at 3 °C for 24 h and then homogenized to below 500 µm using a grinder (PULVERISETTE 11, Fritsch.de, Idar-Oberstein, Germany). Barley (0, 25, 50, 75, and 100%) and rice were cooked with purified water in a 5:6 grains-to-water ratio to determine whether the barley concentration affected the antioxidant activity of the barley nurungji. In the rice cooking step, the grains were cooked using the electric rice cooker (HRC-NMI0601, Cuchen Co., Ltd., Seoul, Republic of Korea) or the electric pressure rice cooker (SRP-H1051FI, Cuckoo Electronics, Yangsan, Republic of Korea) to determine whether the cooking equipment affected the antioxidant activity of the barley nurungji. In the preparation step, the nurungji maker (BE-5200, BETHEL-COOK Inc., Hwaseong, Republic of Korea) was preheated until it reached 205 °C. The barley nurungji was prepared by browning 5 g of cooked grains in a preheated nurungji maker for 3 min at 205 °C. The prepared barley nurungji was homogenized to below 500 µm using a grinder (PULVERISETTE 11, Fritsch, Idar-Oberstein, Germany) and stored in a freezer (KA33-73, Korea Ace Scientific Co., Ltd., Seoul, Republic of Korea) until use.

### 2.3. MRP Solution Preparation

Preparation of MRP solutions has been described previously [25]. D-Glucose solution (5 mL, 0.05 M) and 0.05 M D-Fructose solution (5 mL) were each mixed with 5 mL of 0.05 M L-lysine solution individually at a 1:1 ratio to produce samples without *β*-glucan (GL and FL, respectively). Subsequently, 0.1% *β*-glucan was added to these solutions to produce *β*-glucan-added samples (GLB and FLB, respectively). The mixtures were heated at 100 °C for 0, 2, 4, and 6 h using a heat block (ThermoMixer C, Eppendorf, Hamburg, Germany). After heat treatment, the sample tubes were immediately immersed in ice for cooling and stored in a refrigerator (KA.33-73, Korea Ace Scientific Co., Ltd.).

### 2.4. Physicochemical Properties

The moisture and ash content were measured according to the Association of Official Analytical Collaboration guidelines [26]. Moisture content was measured using the air-oven method at 105 °C with 1 g of sample, and ash content was found using the dry ashing method. The pH of 3 mL of the sample was measured using a pH meter (S220-K, Mettler Toledo International, Inc., Seoul, Republic of Korea). Color measurements were performed by evenly spreading 2 g of sample powder in a Petri dish (35 × 10 mm) using a colorimeter (CR-10 Plus, Konica Minolta Holdings, Inc., Tokyo, Japan), with the white plate having a lightness (L*) value of 97.5, a red/green (a*) value of −0.5, and a yellow/blue (b*) value of 3.0. The color differences (ΔE*) between the control and samples were calculated using the following equation:


ΔE* = [(ΔL*)^2^ + (Δa*)^2^ + (Δb*)^2^]^(1/2)^

The browning intensity was measured at 420 nm using a spectrophotometer (UV-1800, Shimadzu, Tokyo, Japan) with 1 mL of the sample centrifuged at 16,000 rpm for 20 min (Combi 524R, Hanil, Daejeon, Republic of Korea). Total soluble solid and reducing sugar contents were measured using samples centrifuged at 16,000 rpm for 20 min (Combi 524R, Hanil, Daejeon, Republic of Korea). Total soluble solids content was measured using a digital refractometer (SCM-1000, HM Digital Inc., Seoul, Republic of Korea) with 200 μL of sample. The reducing sugar content was determined using the 3,5-dinitrosalicylic acid (DNS) method. Thereafter, 3 mL of the sample was mixed with 3 mL of 1% DNS solution and heated at 90 °C in a water bath for 5 min. Subsequently, 1 mL of 40% Rochelle salt was added, and the mixture was cooled in cold water. Absorbance was measured at 540 nm using the standard curve equation for glucose (mg/g).

### 2.5. Antioxidant Component Analysis and Antioxidant Activity Assay

Centrifugation (Combi 524R, Hanil, Daejeon, Republic of Korea) was performed at 4000 rpm for 5 min and 16,000 rpm for 20 min to measure the antioxidant components and activity of the samples. The total polyphenol content was measured according to the modified method of Arnous et al. [27]. Briefly, 50 μL of Folin–Ciocalteu’s reagent was added to 500 μL of the sample and reacted for 3 min. Subsequently, 1 mL of 2% Na_2_CO_3_ was added and left in the dark for 30 min. Absorbance was measured at 750 nm using a spectrophotometer (UV-1800, Shimadzu) and calculated from a standard curve equation for gallic acid (mg/g). Total flavonoid content was measured using a modified method based on Shen et al. [28]. A total of 75 μL of 5% NaNO_2_ solution was added to 2 mL of the sample and reacted for 5 min, 150 μL of 10% AlCl_3_·6H_2_O was added and reacted for 6 min, and 500 μL of 1 M NaOH was added and left in the dark for 11 min. Absorbance was measured at 415 nm using a spectrophotometer (UV-1800, Shimadzu) and calculated from a standard curve equation for rutin (mg/g). The DPPH radical scavenging activity was measured using a modified method described by Brand-Williams et al. [29]. A 0.2 mM DPPH reagent was prepared and adjusted with methanol to an absorbance value of 1.0. Thereafter, 1 mL of DPPH reagent was added to 100 μL of the sample, and the mixture was left in the dark for 30 min. Absorbance was measured at 517 nm using a spectrophotometer (UV-1800, Shimadzu), and the absorbance was calculated as a percentage using the absorbance of a blank with 100 μL of distilled water and DPPH reagent. The formula was as follows:


DPPH radical scavenging (%) = [(*A*_control_ − *A*_sample_/*A*_control_)] × 100

ABTS radical scavenging activity was measured using a modified method from Re et al. [30]. A mixture of 7 mM ABTS and 2.4 mM potassium persulfate was allowed to react in the dark for 12 h to form ABTS+ (ABTS cation radical), and the absorbance was adjusted with phosphate-buffered saline to 0.7. Subsequently, 1 mL of ABTS+ was added to 100 μL of the sample, and the mixture was left in the dark for 30 min. Absorbance was measured at 735 nm using a spectrophotometer (UV-1800, Shimadzu) and calculated from a standard curve equation for Trolox (mM). The ferric reducing antioxidant power (FRAP) was measured using the method described by Benzie and Strain [31]. A FRAP working solution was prepared by mixing 0.2 M sodium acetate buffer, 10 mM 2,4,6-tripyridyl-S-triazine, 20 mM ferric chloride hexahydrate, and distilled water at a ratio of 10:1:1:1. The solution was then heated at 37 °C for 30 min in a water bath (WCB-22, Daihan Scientific Co., Ltd., Wonju, Republic of Korea). Thereafter, 1 mL of FRAP working solution was added to 100 μL of the sample, and the mixture reacted in the dark for 30 min. Absorbance was measured at 595 nm using a spectrophotometer (UV-1800, Shimadzu) and calculated from a standard curve equation for Trolox (mM). Reducing power was measured using the method described by Canabady-Rochelle et al. [32], with the pH of the 0.2 M sodium phosphate buffer adjusted to 6.6. Briefly, 300 μL of 0.2 M sodium phosphate buffer and 300 μL of 1% potassium ferricyanide were added to 100 μL of the sample, and the solution was reacted at 50 °C for 20 min in a water bath (WCB-22, Daihan Scientific Co., Ltd.). Then, 300 μL of 10% trichloroacetic acid and 100 μL of 0.1% ferric chloride were added. Absorbance was measured at 700 nm using a spectrophotometer (UV-1800, Shimadzu) and calculated from a standard curve equation for Trolox (mM).

### 2.6. Sensory Evaluation

The consumer acceptance test was performed in compliance with the guidelines of the Wonkwang University Institutional Review Board. One hundred and four consumers, aged 20–30 years, participated in the consumer acceptance test. Participants interested in nurungji without any existing food allergies were recruited through email and flyers. All the participants were asked to complete a consent form before the sensory evaluation. The acceptance tests were conducted in the sensory booth of the sensory evaluation laboratory. Sensory evaluations were conducted at 10 a.m. and 3 p.m. for 40 min. Before the sensory evaluation, the evaluation procedure and rinsing methods were explained to the participants. The nurungji sample was served at room temperature, and 5 g of the sample was placed in three-digit coded sensory cups (7 × 3 cm) along with bottled water to rinse the mouth during the evaluation. Each sensory cup was labeled with a random three-digit number extracted from a random number table, and all samples were provided in a balanced order based on a mutually orthogonal Latin square design. After completing a general characteristics questionnaire, participants were asked to evaluate five nurungji samples blended with barley (0, 25, 50, 75, and 100%) for overall acceptance, appearance acceptance, aroma acceptance, taste acceptance, sweetness intensity, texture acceptance, savory intensity, aftertaste acceptance, and purchase intention. Participants used a nine-point hedonic scale anchored on 1 = dislike extremely, 5 = neither like nor dislike, and 9 = like extremely. Purchase intent was scaled using a five-point Likert scale anchored with 1 = definitely would not purchase, 3 = may or may not purchase, and 5 = would definitely purchase.

### 2.7. Statistical Analysis

Measurement results were expressed as the mean ± standard deviation using XLSTAT (Lurnivero, Denver, CO, USA). Differences between sample measurement values for physicochemical characteristics and antioxidant properties were assessed using analysis of variance (ANOVA) at a significance level of 0.05. Duncan’s multiple range test was used to verify significant differences between mean values at a significance level of 0.05. Consumer test ratings of overall acceptance, appearance acceptance, aroma acceptance, taste acceptance, sweetness intensity, texture acceptance, savory intensity, aftertaste acceptance, and purchase intent for each sample were analyzed using ANOVA. The mean separation was determined using Fisher’s least significant difference at a significance level of 0.05. Data visualization was achieved using principal component analysis (PCA), and a matrix of 25 variables for the five nurungji samples was analyzed.

## 3. Results and Discussion

### 3.1. Physicochemical Characteristics of Barley Nurungji

The physicochemical characteristics of barley nurungji prepared using different cooking equipment, barley varieties, and barley blending ratios are presented in Table 1.

The moisture content of barley nurungji ranged from 3.38 to 6.96%, and the moisture content markedly decreased as the addition of barley increased. The moisture content in waxy barley nurungji (WBN-EC and WBN-PC) was significantly higher (*p* < 0.05) than in hulled barley nurungji (HBN-EC and HBN-PC). Notably, the moisture content in WBN-EC was markedly higher than that in WBN-PC. Therefore, the moisture content of barley nurungji may be affected not only by the water absorption of amylose and amylopectin in waxy barley granules, but also by the mechanical strength of the electric pressure rice cooker. Biduski et al. [33] demonstrated that high amylose starches change their microstructure under higher mechanical pressure, resulting in firmer and stronger gels. The crude ash content of the barley nurungji significantly increased as the barley concentration increased and was higher with hulled barley than with waxy barley. Lukinac and Jukic [34] revealed that hulled barley has a higher ash content than that in hull-less barley; however, cultivar characteristics and cultivation environment influence the chemical composition of barley. The total soluble solid content and reducing sugar content of barley nurungji increased with the addition of barley, with the total soluble solid content ranging from 0.48 to 2.30° Brix and reducing sugar content ranging from 32.41 to 95.29 G.E. mg/g, both showing significant increases in the WBN-PC group (*p* < 0.05). Girhammar and Nair [35] showed that barley contains relatively high amounts of glucose and other monosaccharides, such as xylose and mannose. Lukinac and Jukic [34] reported that hull-less barley has a higher sugar content than that in hulled barley. Chromaticity measurements revealed that the L* value of barley nurungji significantly decreased as barley content inclined, whereas the a* and b* values increased. Boyd et al. [36] showed that barley lightness decreased with heat treatment, whereas redness and yellowness increased, which is consistent with the results of this study. In terms of hulled barley and waxy barley, nurungji blended with hulled barley showed significantly higher values in L* and b* values than that in nurungji blended with waxy barley. In terms of the degree of color difference, the ΔE value increased as barley was added compared with that in the control nurungji (BN0) under the same electric cooker. WBN-PC showed the highest value, followed by WBN-EC, hulled barley nurungji cooked using an electric pressure rice cooker (HBN-PC), and hulled barley nurungji cooked using an electric rice cooker (HBN-EC). These results demonstrate that the color differences between the barley nurungji samples were markedly different, with colors of different shades in all barley nurungji samples. The browning intensity of barley nurungji ranged from 0.10 to 1.31, showing a significant increase with the addition of barley (*p* < 0.05). WBN-PC showed the highest browning intensity, followed by WBN-EC, HBN-PC, and HBN-EC. The browning of barley when cooked is related to phenolic compounds and the Maillard reaction [37,38].

### 3.2. Antioxidant Contents and Antioxidant Activities of Barley Nurungji

**Figure 2:**
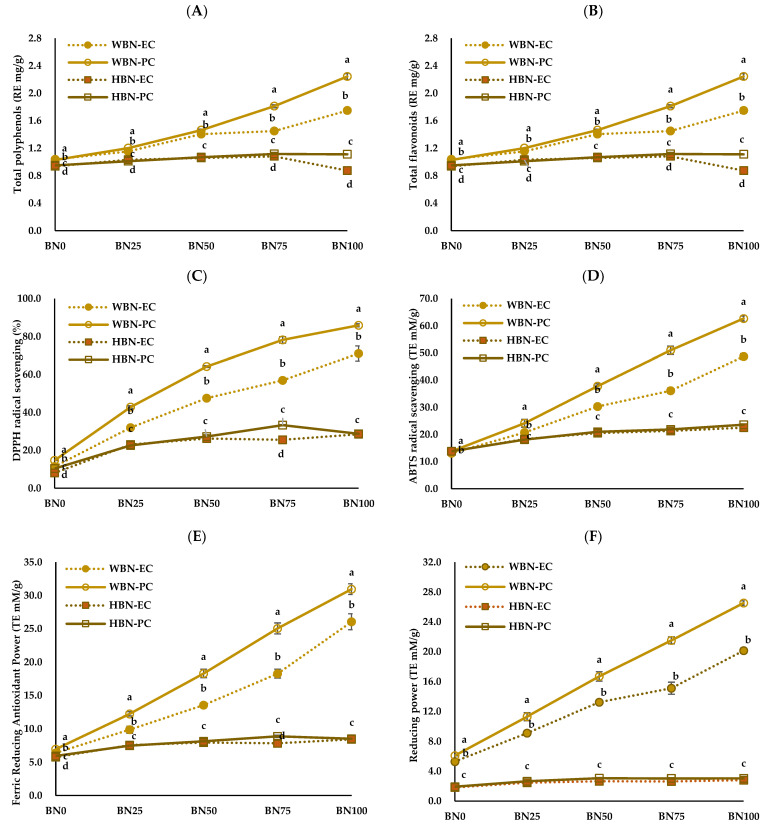
Antioxidant contents (**A**,**B**) and antioxidant activities (**C**–**F**) of barley nurungji samples using different types of cooking equipment, barley varieties, and barley addition ratios. Waxy barley nurungji cooked using an electric rice cooker (WBN-EC); waxy barley nurungji cooked using an electric pressure rice cooker (WBN-PC); hulled barley nurungji cooked using an electric rice cooker (HBN-EC); hulled barley nurungji cooked using an electric pressure rice cooker (HBN-PC); BN0, BN25, BN50, BN75, and BN100 indicate different barley addition ratios of 0, 25, 50, 75, and 100%, respectively. Mean values with different letters (a–d) in the same concentration are significantly different at *p* < 0.05.

The antioxidant properties of the barley nurungji are shown in Figure 2. The total polyphenol content ranged from 2.18 to 7.89 G.A. mg/g and increased with the addition of barley (Figure 2A). The total polyphenol content of WBN-PC and WBN-EC significantly increased in a barley concentration-dependent manner, whereas that of HBN-PC and HBN-EC slightly increased.

Holtekjølen and Knutsen [38] demonstrated that barley contains various phenolics, mainly ferulic acid and p-coumaric acid, and that hull-less barley contains more antioxidants. According to Kim et al. [39], the primary phenolic acids in colored barley are phloroglucinol, chlorogenic acid, pyrogallol, and salicylic acid, and the phenolic acid content is higher in hull-less barley than in hulled barley. The total flavonoid content of barley nurungji significantly increased with the amount of added barley (*p* < 0.05) (Figure 2B). The total flavonoid content of WBN-PC and HBN-PC was significantly higher than that of WBN-EC and HBN-EC (*p* < 0.05). The DPPH radical scavenging activity of barley nurungji ranged from 8.05 to 14.72% in BN0 and increased to a maximum of 85.9% with the addition of barley (Figure 2C). The DPPH radical scavenging activity in HBN-PC and WBN-PC was significantly higher than in HBN-EC and WBN-EC (*p* < 0.05). According to He et al. [40], the total polyphenol and flavonoid contents of deodeok extract also increased as the pressure level increased. Verardo et al. [41] reported that the antioxidant properties of bread crust were better than those of dough, which was explained by the Maillard reaction. The antioxidant properties of bread increased with the addition of grains. The ABTS radical scavenging activity was significantly higher in the 100% barley ratio (BN100) group of WBN-PC (Figure 2D). The antioxidant activities of the FRAP assay and reducing power were used to evaluate the reduction of ferric iron (Fe^3+^) to ferrous iron (Fe^2+^) under acidic and neutral pH conditions, respectively. The FRAP assay results and reducing power of WBN-PC and HBN-PC were significantly higher than that of WBN-EC and HBN-EC (Figure 2E,F). Bai et al. [42] researched that free phenolics, *β*-glucan, and antioxidant activity increased with the heat treatment of highland barley. Furthermore, the antioxidant properties of food increase with pressure steaming compared to regular steaming [43]. This is thought to be a result of the differences in methods that promote the conversion of bound phenolics to their free forms [44]. Hull-less barley has relatively higher antioxidant properties than those in hulled barley due to its higher content of *β*-glucan and dietary fiber [45].

### 3.3. Physicochemical Characteristics of the Model MRP Solution

The physicochemical properties of the model MRP solution, which was prepared by heating GL, FL, GLB, and FLB for 0–6 h, are shown in Table 2.

The total soluble solids content of the MRP solutions did not change significantly in the GL and FL samples over the heating period. However, in the GLB and FLB samples, the total soluble solids content significantly increased as heating time increased (*p* < 0.05). Kim and Lee [46] demonstrated that the sugar content of MRP solutions increased as the heating time increased from 1 to 2 h and that the sugar content of the fructose–lysine mixture was higher than that of the glucose–lysine mixture. This result indicates a similarity, where the increase in the total soluble solids content with heating time is attributed to the formation of intermediates and compounds via the Maillard reaction. The higher total soluble solids content in the MRP solutions with *β*-glucan is believed to be a result of the additional breakdown of the water-soluble dietary fiber *β*-glucan [47]. The reducing sugar content of all MRP solutions significantly decreased as the heating time increased (*p* < 0.05). The Lobry de Bruyn–Alberda van Eckenstein transformation, also known as the enolization reaction, allows glucose and fructose to interconvert [48]. In the initial stages of the non-enzymatic browning Maillard reaction, the condensation of sugar and amine results in the formation of glycosylamine, which then undergoes Amadori rearrangement to ketoamine, whereas fructose undergoes Heyns rearrangement to form 2-amino-2-deoxyaldose [49]. The decrease in the reducing sugar content with increasing heating time in this study is thought to be a result of glucose and fructose consumption during the condensation of sugar and amine in the initial stages of the Maillard reaction. The pH of the MRP significantly decreased as heating time increased (*p* < 0.05). Abrantes et al. [50] showed that gallic acid content decreased as the reaction progressed in a model system, which was attributed to its participation in the non-enzymatic browning reaction rather than hydrolysis. In the chromaticity measurement results, the L* value of the MRP significantly decreased with increasing heating time (*p* < 0.05), and the FLB solution had a lower L* value than that of the GLB solution. The a* and b* values showed a significant increase as heating time increased (*p* < 0.05), and the FLB solution had higher values than those of the GLB solution. These findings are similar to those of Kiits and Hu [51], who revealed a decrease in lightness and an increase in redness with heating of MRPs, and a study by Sun et al. [52], who demonstrated that the lightness of fructose MRPs was lower, whereas the redness and yellowness were higher than those of glucose MRPs. In this study, the browning intensity of the MRP solutions with and without *β*-glucan had a significant increase within the same treatment group as heating time increased (*p* < 0.05). The browning intensity of the GLB and FLB solutions was significantly higher than that in the MRPs without *β*-glucan (GL and FL) (*p* < 0.05). Hwang et al. [53] reported that an MPRs solution of fructose-amino acids had higher antioxidant activity than that of glucose-amino acid compounds, which was attributed to the higher content of browning precursor substances, depending on the type of reducing sugar. The results of this study are consistent with those of Kwak and Lim [54], who observed an increase in the browning intensity of the MRP solutions formed between glucose and various amino acids such as aspartic acid, alanine, valine, serine, and cysteine as heating time increased. Therefore, the occurrence of non-enzymatic browning reactions due to heating influences changes in ingredients, such as glucose, fructose, and *β*-glucan.

### 3.4. Antioxidant Activities of MRPs

**Figure 3:**
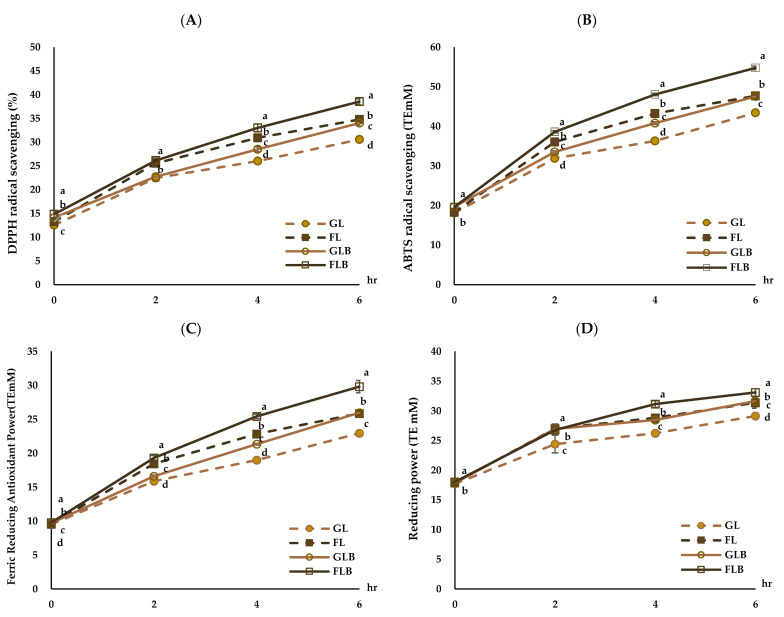
Antioxidant activities of model MRP solutions (**A**–**D**); glucose–lysine (GL), fructose–lysine (FL), glucose–lysine+*β*-glucan (GLB), fructose–lysine+*β*-glucan (FLB). Mean values with different letters (a–d) at the same time are significantly different at *p* < 0.05.

The antioxidant activities of the MRPs are shown in Figure 3. The DPPH radical scavenging activity of the MRP solutions significantly increased with heating time (*p* < 0.05), and the DPPH radical scavenging activity in the groups with *β*-glucan (GLB, FLB) was significantly higher than that in the groups without *β*-glucan (GL, FL) (Figure 3A). The DPPH radical scavenging activity ranged from 12.53 to 30.58% in GL, 13.27 to 34.79% in FL, 14.12 to 34.03% in GLB, and 14.83 to 38.56% in FLB, with FLB showing significantly higher DPPH radical scavenging activity (*p* < 0.05). These results align with those of Hwang et al. [53], who found that the DPPH radical scavenging activity of fructose–amino acid model systems, except for cysteine, was higher than that of glucose–amino acid model systems in all sugar–amino acid mixtures analyzed. In this study, the increase in antioxidant activity with heating time in the groups with *β*-glucan (GLB, FLB) was significantly higher than that in the groups without *β*-glucan (GL, FL) (*p* < 0.05). This change may be related to the breaking down of *β*-glucan during heating and the use of reducing sugars during the non-enzymatic browning reaction. The ABTS radical scavenging activity of the MRP solutions significantly increased as heating time increased (*p* < 0.05), and the ABTS radical scavenging activity in the *β*-glucan-added groups (GLB, FLB) was significantly higher than that in the *β*-glucan-free groups (GL, FL) (Figure 3B). The ABTS radical scavenging activity of FLB was the highest, followed by FL, GLB, and GL. Liu et al. [55] reported that the Maillard reaction is influenced by factors such as amino acid type, sugars, pH value, temperature, and time, non-enzymatic intermediates, volatile compounds, the color pigment melanoidin, and antioxidant properties.

The FRAP of the MRP solutions also significantly increased with heating time, and the FRAP of the groups with *β*-glucan (GLB, FLB) was significantly higher than that of the groups without *β*-glucan (GL, FL) (Figure 3C). The highest FRAP was measured in FLB, followed by FL, GLB, and GL. Kim and Lee [56] showed that the FRAP of fructose/L-lysine MRP solution was higher than that of glucose/L-lysine MRP solution over heating time. The FRAP assay involves the reduction of ferric iron to ferrous iron in the presence of an antioxidant solution. As the non-enzymatic browning reaction progresses, the reducing power of MRPs of all samples also increases with heating time (Figure 3D). The reducing power of MRPs in the groups with *β*-glucan (GLB, FLB) was higher than that in the groups without *β*-glucan (GL, FL), ranging from 17.86 to 33.11 T.E. mM (*p* < 0.05), with FLB showing significantly higher reducing power (*p* < 0.05). Chung et al. [57] demonstrated that the reducing power of MRPs increased with heating time and continued to increase after 18 h of heating but slightly decreased after 48 h. This study confirmed that the increase in antioxidant activities of nurungji blended with barley could be closely related to the *β*-glucan content of barley.

### 3.5. Consumer Acceptance of Barley Nurungji

**Figure 4:**
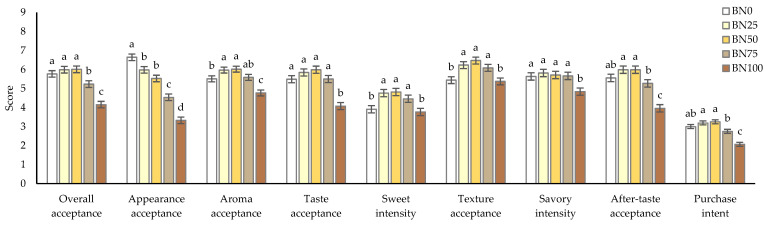
Consumer acceptance test (*n* = 104) of nurungji blended with waxy barley using an electric pressure rice cooker: nurungji (BN0, BN25, BN50, BN75, BN100) prepared with different barley addition ratios (0, 25, 50, 75, and 100%, respectively). Mean values with different letters are significantly different at *p* < 0.05.

A consumer acceptance test was performed to clarify the optimal concentration of barley in barley nurungji from a consumer choice perspective. The consumer acceptance test results are shown in Figure 4. The sample groups prepared using an electric pressure rice cooker with varying barley ratios (BN0, 25% barley ratio [BN25], 50% barley ratio [BN50], 75% barley ratio [BN75], and BN100) demonstrated high antioxidant properties; hence, they were selected for the consumer acceptance test.

The evaluation was conducted using a nine-point hedonic scale to assess overall acceptance, appearance acceptance, aroma acceptance, taste acceptance, sweetness intensity, texture acceptance, savory intensity, and aftertaste acceptance. The overall acceptance of BN50 had the highest score of 6.01 points. Overall acceptance increased from BN0 to BN50 without a significant difference and decreased significantly from BN75. This suggests that barley addition below 75% positively affected the overall acceptance of barley nurungji. The appearance acceptance for barley nurungji significantly decreased with the addition of barley. This may be because of the browning effect on the appearance of barley nurungji. Aroma acceptance increased with the addition of barley, the highest at BN50, with a score of 6.02 points. Notably, BN100 showed a significantly lower aroma acceptance compared to BN0. Taste acceptance also increased with the addition of barley, peaking at BN50. Although no significant differences were found between the BN0 and BN75 samples, BN100 had a significantly lower taste acceptance (*p* < 0.05). These data indicated that the addition of an appropriate amount of barley positively affected the taste acceptance of barley nurungji. The sweetness intensity of barley nurungji was the highest in BN50, whereas it was significantly lower in BN0 and BN100 (*p* < 0.05). Texture and aftertaste acceptance showed a trend similar to that of sweetness intensity (*p* < 0.05). The purchase intent for barley nurungji that was evaluated using a five-point category scale was highest for BN50 at 3.25 points and significantly lower for BN75 and BN100 (*p* < 0.05). Consumer acceptance evaluation indicated that BN50 had the highest scores for overall acceptance, aroma acceptance, taste acceptance, sweetness intensity, texture acceptance, aftertaste acceptance, and purchase intent. However, when the barley content was 75% or higher, the overall acceptance was significantly decreased. According to Hęś et al. [21], adding barley to bread imparts bitterness, which is attributed to phenolic acids and flavanol polymers. Adding an appropriate amount of barley positively affects the quality characteristics of nurungji; on the contrary, exceeding a certain amount negatively affects its quality.

**Figure 5:**
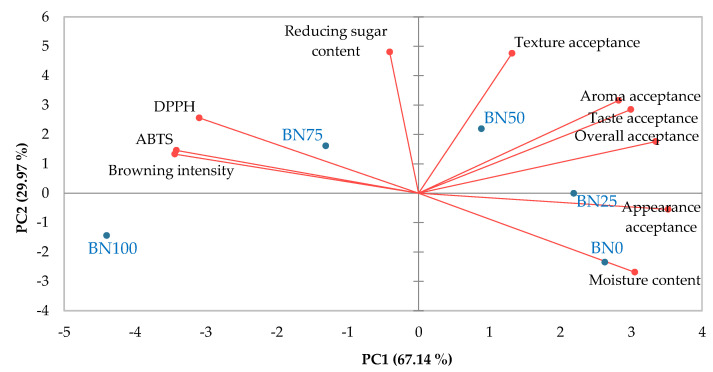
Biplot representation of the principal component analysis of physicochemical properties, antioxidant activities, and consumer acceptance of barley nurungji. An evaluation test was performed by 104 consumers. BN0, BN25, BN50, BN75, and BN100 indicate different barley addition ratios of 0, 25, 50, 75, and 100%, respectively.

The data were visualized using PCA to examine the correlation between barley nurungji and its quality properties, including physicochemical characteristics, antioxidant activities, and consumer acceptance (Figure 5). The score plot defined by the two principal components (PC1 and PC2) accounted for 67.14% and 29.97% of variability, respectively. They showed a clear separation among the BN samples. BN0, BN25, and BN50 were PC1 positive, whereas BN75 and BN100 were PC1 negative. BN50 showed highly positive PC1 and PC2 values in quadrant I of the plot, characterized by overall acceptance, taste and aroma acceptance, and texture acceptance. BN0 and BN25, located in the fourth quarter, were characterized by appearance acceptance and moisture content. BN75, located in the second quarter, was characterized by antioxidant activities (ABTS and DPPH) and browning intensity. Therefore, barley nurungji with low barley content is characterized by moisture and appearance. Barley nurungji with a 50% barley blend was highly related to acceptance characteristics, and when barley addition exceeded 75%, there was a close relationship between antioxidants and browning intensity.

## 4. Conclusions

This study effectively showed that the antioxidant activities (DPPH, ABTS, FRAP, and RP) of barley nurungji significantly increased (1) with waxy barley than those of hulled barley and (2) with an electric pressure rice cooker than those of an electric rice cooker. The increase in the antioxidant activities of nurungji may be explained by the increased amount of antioxidant compounds, such as total polyphenols and total flavonoids, which were released from the matrix through thermal processing and non-enzymatic browning reactions. Another reason could be the presence of other phytochemicals, such as *β*-glucan in barley. In the MRP model system with *β*-glucan, the antioxidant activities (DPPH, ABTS, FRAP, and reducing power) of the MRP solutions with *β*-glucan significantly increased compared to those of the MRP solution without *β*-glucan. In conclusion, the preparation of nurungji blended with waxy barley using an electric pressure rice cooker improved the quality characteristics of nurungji because the addition of barley increased the reducing sugar content, browning intensity, antioxidant components, and antioxidant activities. The *β*-glucan in barley affected the antioxidant activity of non-enzymatic browning reaction products. The consumer acceptance evaluation indicated that a 50% barley addition ratio was the most appropriate, as it yielded significantly higher scores for overall acceptance, aroma acceptance, taste acceptance, sweetness intensity, texture acceptance, aftertaste acceptance, and purchase intent. The results of this study broaden existing understanding of the relationship between the quality characteristics and consumer acceptance of barley nurungji. This study did not evaluate the correlation between sensory acceptability and texture profile analysis in relation to the physicochemical properties, which should be addressed in future studies. Although barley is not commonly used in foods, this study demonstrated that the addition of barley had an overall positive effect on nurungji. This suggests that additional studies on barley should be conducted to increase its application in processed gluten-free foods.

## Figures and Tables

**Table 1:** Effects of cooking equipment, barley varieties, and blending ratio of barley on physicochemical quality of barley nurungji.

		Barley Ratio (%)	HBN-EC	HBN-PC	WBN-EC	WBN-PC
Moisture content (%)		BN0	4.16 ± 0.03 ^aC^	4.71 ± 0.04 ^aD^	6.96 ± 0.02 ^aA^	6.69 ± 0.03 ^aB^
		BN25	3.89 ± 0.05 ^bD^	4.49 ± 0.08 ^bC^	6.02 ± 0.04 ^bA^	5.69 ± 0.03 ^bB^
		BN50	3.85 ± 0.04 ^bD^	4.36 ± 0.06 ^cB^	4.99 ± 0.04 ^cA^	4.20 ± 0.04 ^cC^
		BN75	4.11 ± 0.04 ^aB^	3.47 ± 0.03 ^dD^	4.31 ± 0.04 ^dA^	3.82 ± 0.02 ^dC^
		BN100	3.38 ± 0.21 ^cB^	3.41 ± 0.04 ^dB^	4.14 ± 0.04 ^eA^	3.44 ± 0.03 ^eB^
Crude ash content (%)		BN0	0.25 ± 0.04 ^dA^	0.24 ± 0.02 ^dA^	0.16 ± 0.01 ^eB^	0.17 ± 0.02 ^eB^
		BN25	0.39 ± 0.04 ^cA^	0.28 ± 0.05 ^dB^	0.32 ± 0.04 ^dB^	0.33 ± 0.03 ^dAB^
		BN50	0.53 ± 0.03 ^bA^	0.40 ± 0.01 ^cB^	0.54 ± 0.03 ^cA^	0.56 ± 0.01 ^cA^
		BN75	0.55 ± 0.04 ^bC^	0.55 ± 0.02 ^bC^	0.68 ± 0.02 ^bB^	0.85 ± 0.02 ^bA^
		BN100	0.77 ± 0.02 ^aC^	0.66 ± 0.04 ^aD^	0.88 ± 0.02 ^aB^	1.07 ± 0.01 ^aA^
Total soluble solids (°Brix)		BN0	0.48 ± 0.05 ^cC^	0.68 ± 0.03 ^eA^	0.57 ± 0.05 ^dB^	0.60 ± 0.05 ^dB^
		BN25	0.86 ± 0.05 ^bC^	0.88 ± 0.05 ^dC^	1.28 ± 0.05 ^cB^	1.60 ± 0.04 ^cA^
		BN50	1.03 ± 0.060 ^aC^	1.02 ± 0.03 ^cC^	1.88 ± 0.05 ^bB^	2.20 ± 0.06 ^bA^
		BN75	1.03 ± 0.04 ^aD^	1.22 ± 0.02 ^aC^	1.85 ± 0.10 ^bB^	2.20 ± 0.05 ^bA^
		BN100	1.03 ± 0.05 ^aC^	1.13 ± 0.01 ^bB^	2.28 ± 0.05 ^aA^	2.30 ± 0.09 ^aA^
Reducing sugar (mg/g)		BN0	32.62 ± 0.17 ^bB^	32.41 ± 0.24 ^cB^	33.07 ± 0.05 ^eA^	32.89 ± 0.00 ^dA^
		BN25	33.14 ± 0.24 ^aC^	32.72 ± 0.16 ^cC^	58.14 ± 0.12 ^dB^	76.55 ± 0.08 ^cA^
		BN50	33.22 ± 0.09 ^abD^	37.19 ± 0.28 ^bC^	87.30 ± 0.46 ^cB^	90.76 ± 0.16 ^bA^
		BN75	33.48 ± 0.28 ^aD^	38.38 ± 0.44 ^aC^	89.56 ± 0.25 ^bB^	90.76 ± 0.72 ^bA^
		BN100	33.07 ± 0.12 ^bC^	32.74 ± 0.20 ^cC^	91.51 ± 0.08 ^aB^	95.29 ± 0.05 ^aA^
pH		BN0	5.30 ± 0.04 ^bD^	5.56 ± 0.04 ^aB^	5.67 ± 0.02 ^aA^	5.49 ± 0.02 ^aC^
		BN25	5.32 ± 0.03 ^bB^	5.31 ± 0.01 ^cBC^	5.40 ± 0.02 ^bA^	5.29 ± 0.01 ^bC^
		BN50	5.30 ± 0.01 ^bC^	5.34 ± 0.01 ^bcB^	5.36 ± 0.01 ^cA^	5.18 ± 0.00 ^cD^
		BN75	5.37 ± 0.01 ^aA^	5.34 ± 0.01 ^bA^	5.19 ± 0.02 ^dB^	5.06 ± 0.01 ^dC^
		BN100	5.39 ± 0.01 ^aA^	5.36 ± 0.01 ^bB^	5.16 ± 0.02 ^eC^	5.07 ± 0.01 ^dD^
Color	L* value	BN0	86.68 ± 0.15 ^aA^	86.90 ± 0.24 ^aA^	83.03 ± 0.24 ^aB^	82.90 ± 0.29 ^aB^
		BN25	76.73 ± 0.46 ^bB^	77.43 ± 0.15 ^bA^	74.35 ± 0.24 ^bC^	72.93 ± 0.43 ^bD^
		BN50	74.68 ± 0.59 ^dA^	75.20 ± 0.28 ^cA^	71.08 ± 1.28 ^cB^	66.10 ± 0.96 ^cC^
		BN75	75.70 ± 0.70 ^cA^	73.35 ± 0.24 ^dB^	67.33 ± 0.46 ^dC^	61.10 ± 0.36 ^dD^
		BN100	73.03 ± 0.62 ^eB^	75.03 ± 0.49 ^cA^	63.88 ± 0.42 ^eC^	59.73 ± 0.79 ^eD^
	a* value	BN0	4.73 ± 0.13 ^dB^	4.73 ± 0.13 ^dB^	5.95 ± 0.10 ^cA^	6.08 ± 0.13 ^dA^
		BN25	7.60 ± 0.22 ^abA^	7.08 ± 0.10 ^bcB^	7.13 ± 0.22 ^bB^	6.73 ± 0.17 ^cC^
		BN50	7.75 ± 0.19 ^aA^	7.18 ± 0.21 ^bB^	7.28 ± 0.36 ^bB^	7.98 ± 0.21 ^aA^
		BN75	6.95 ± 0.39 ^cC^	7.50 ± 0.16 ^aD^	7.90 ± 0.24 ^aA^	7.95 ± 0.10 ^abA^
		BN100	7.35 ± 0.30 ^bC^	6.87 ± 0.06 ^cD^	8.03 ± 0.05 ^aA^	7.73 ± 0.13 ^bB^
	b* value	BN0	14.95 ± 0.45 ^aB^	15.53 ± 0.29 ^cA^	14.90 ± 0.14 ^cB^	14.85 ± 0.17 ^bB^
		BN25	17.40 ± 0.39 ^aA^	16.43 ± 0.36 ^bB^	15.38 ± 0.29 ^abC^	14.15 ± 0.24 ^cD^
		BN50	17.43 ± 0.33 ^aA^	16.43 ± 0.64 ^bA^	15.35 ± 0.13 ^bA^	15.28 ± 0.28 ^aA^
		BN75	17.13 ± 0.62 ^aA^	17.28 ± 0.43 ^aA^	15.68 ± 0.32 ^aB^	14.23 ± 0.15 ^cC^
		BN100	17.28 ± 0.28 ^aA^	17.33 ± 0.24 ^aA^	15.50 ± 0.08 ^abB^	14.15 ± 0.41 ^cC^
	ΔE*	BN0	17.07 ± 0.40 ^cB^	17.29 ± 0.37 ^eB^	20.13 ± 0.19 ^eA^	20.24 ± 0.35 ^eA^
		BN25	26.94 ± 0.61 ^bC^	25.73 ± 0.17 ^dD^	27.88 ± 0.37 ^dB^	28.55 ± 0.46 ^dA^
		BN50	27.87 ± 0.57 ^bC^	27.62 ± 0.29 ^cC^	30.79 ± 1.27 ^cB^	35.44 ± 0.88 ^cA^
		BN75	27.46 ± 0.98 ^bD^	29.66 ± 0.32 ^aC^	34.43 ± 0.54 ^bB^	39.77 ± 0.34 ^bA^
		BN100	29.89 ± 0.73 ^aC^	28.09 ± 0.36 ^bD^	37.56 ± 0.37 ^aB^	41.00 ± 0.63 ^aA^
Browning intensity		BN0	0.11 ± 0.01 ^dB^	0.14 ± 0.01 ^dA^	0.10 ± 0.00 ^eC^	0.10 ± 0.00 ^eC^
		BN25	0.15 ± 0.03 ^cC^	0.15 ± 0.02 ^bD^	0.23 ± 0.01 ^dB^	0.35 ± 0.00 ^dA^
		BN50	0.17 ± 0.02 ^bC^	0.15 ± 0.01 ^cD^	0.29 ± 0.01 ^cB^	0.69 ± 0.01 ^cA^
		BN75	0.15 ± 0.01 ^cD^	0.19 ± 0.00 ^aC^	0.36 ± 0.00 ^bB^	0.96 ± 0.01 ^bA^
		BN100	0.19 ± 0.03 ^aC^	0.19 ± 0.02 ^aC^	0.76 ± 0.02 ^aB^	1.31 ± 0.00 ^aA^

Mean values within each column (^a–e^) and each row (^A–D^) with different superscript letters are significantly different at *p* < 0.05; hulled barley nurungji cooked using an electric rice cooker (HBN-EC), hulled barley nurungji cooked using an electric pressure rice cooker (HBN-PC), waxy barley nurungji cooked using an electric rice cooker (WBN-EC), waxy barley nurungji cooked using an electric pressure rice cooker (WBN-PC); L*, lightness; a*, redness; b*, yellowness; ΔE*, color differences. BN0, BN25, BN50, BN75, and BN100 indicate different barley addition ratios of 0, 25, 50, 75, and 100%, respectively.

**Table 2:** Physicochemical characteristics of the model MRP solutions prepared with glucose–lysine (GL), fructose–lysine (FL), glucose–lysine–*β*-glucan (GLB), and fructose–lysine–*β*-glucan (FLB) at various heating times.

		Heating Time (h)	GL	FL	GLB	FLB
Total soluble solids (°Brix)		0	1.10 ± 0.03 ^aA^	1.10 ± 0.03 ^aA^	1.10 ± 0.03 ^cA^	1.10 ± 0.03 ^cA^
		2	1.10 ± 0.02 ^aB^	1.10 ± 0.02 ^aB^	1.20 ± 0.02 ^bA^	1.20 ± 0.02 ^bA^
		4	1.10 ± 0.03 ^aB^	1.10 ± 0.03 ^aB^	1.20 ± 0.03 ^bA^	1.20 ± 0.03 ^bA^
		6	1.10 ± 0.03 ^aB^	1.10 ± 0.03 ^aB^	1.30 ± 0.03 ^aA^	1.30 ± 0.03 ^aA^
Reducing sugar (mg/g)		0	44.55 ± 0.65 ^aD^	47.54 ± 0.28 ^aC^	50.06 ± 1.01 ^aB^	53.67 ± 1.29 ^aA^
		2	38.07 ± 0.83 ^bB^	36.36 ± 0.74 ^bC^	43.91 ± 1.95 ^bA^	42.17 ± 2.03 ^bA^
		4	33.41 ± 0.65 ^cB^	30.26 ± 0.61 ^cC^	37.99 ± 0.73 ^cA^	37.16 ± 1.09 ^cA^
		6	29.78 ± 0.79 ^dC^	28.69 ± 0.77 ^dD^	36.93 ± 1.71 ^cA^	34.43 ± 1.72 ^dB^
pH		0	10.13 ± 0.02 ^aB^	10.12 ± 0.03 ^aB^	10.26 ± 0.02 ^aA^	10.28 ± 0.02 ^aA^
		2	9.22 ± 0.07 ^bC^	9.22 ± 0.02 ^bBC^	9.43 ± 0.03 ^bA^	9.28 ± 0.03 ^bB^
		4	8.70 ± 0.05 ^cB^	8.68 ± 0.01 ^cB^	8.90 ± 0.01 ^cA^	8.73 ± 0.07 ^cB^
		6	8.31 ± 0.02 ^dB^	8.15 ± 0.02 ^dD^	8.52 ± 0.01 ^dA^	8.25 ± 0.01 ^dC^
Color	L* value	0	58.23 ± 0.13 ^aA^	58.20 ± 0.18 ^aA^	57.35 ± 0.37 ^aB^	57.55 ± 0.26 ^aB^
		2	53.53 ± 0.33 ^bA^	51.63 ± 0.46 ^bC^	52.28 ± 0.32 ^bB^	50.30 ± 0.47 ^bD^
		4	49.98 ± 0.30 ^cA^	47.63 ± 0.46 ^cC^	48.50 ± 0.63 ^cB^	47.65 ± 0.99 ^cBC^
		6	47.88 ± 0.64 ^dA^	46.93 ± 0.13 ^dB^	45.33 ± 0.78 ^dC^	43.88 ± 0.57 ^dD^
	a* value	0	−0.60 ± 0.03 ^dA^	−0.60 ± 0.03 ^dA^	−0.60 ± 0.03 ^dA^	−0.60 ± 0.03 ^dA^
		2	0.90 ± 0.18 ^cD^	2.23 ± 0.15 ^cB^	1.63 ± 0.26 ^cC^	3.15 ± 0.06 ^cA^
		4	3.48 ± 0.05 ^bD^	5.00 ± 0.00 ^bB^	4.18 ± 0.35 ^bC^	5.45 ± 0.40 ^bA^
		6	5.38 ± 0.33 ^aC^	6.55 ± 0.13 ^aB^	6.85 ± 0.30 ^aB^	7.95 ± 0.19 ^aA^
	b* value	0	−2.50 ± 0.08 ^dC^	−1.68 ± 0.26 ^dA^	−1.65 ± 0.33 ^dA^	−2.10 ± 0.20 ^dB^
		2	12.80 ± 0.47 ^cC^	15.00 ± 0.35 ^cB^	13.23 ± 0.78 ^cC^	16.53 ± 0.13 ^cA^
		4	16.85 ± 0.40 ^bC^	17.58 ± 0.15 ^bB^	16.75 ± 0.31 ^bC^	17.93 ± 0.05 ^aA^
		6	18.55 ± 0.13 ^aB^	18.85 ± 0.13 ^aA^	18.13 ± 0.05 ^aC^	17.53 ± 0.38 ^bD^
	ΔE*	0	32.59 ± 0.13 ^dB^	32.69 ± 0.21 ^dB^	33.54 ± 0.40 ^dA^	33.30 ± 0.28 ^dA^
		2	41.32 ± 0.49 ^cD^	44.06 ± 0.27 ^cB^	42.6 6 ± 0.64 ^cC^	45.99 ± 0.44 ^cA^
		4	46.46 ± 0.19 ^bC^	49.00 ± 0.35 ^bA^	47.77 ± 0.73 ^bB^	49.20 ± 0.92 ^bA^
		6	49.29 ± 0.61 ^aD^	50.40 ± 0.16 ^aC^	51.51 ± 0.69 ^aB^	52.68 ± 0.39 ^aA^
Browning intensity		0	0.00 ± 0.00 ^dC^	0.00 ± 0.00 ^dC^	0.01 ± 0.00 ^dB^	0.01 ± 0.00 ^dA^
		2	0.20 ± 0.01 ^cD^	0.27 ± 0.00 ^cB^	0.23 ± 0.02 ^cC^	0.31 ± 0.02 ^cA^
		4	0.34 ± 0.01 ^bC^	0.42 ± 0.01 ^bB^	0.41 ± 0.03 ^bB^	0.46 ± 0.03 ^bA^
		6	0.48 ± 0.02 ^aD^	0.52 ± 0.01 ^aC^	0.57 ± 0.03 ^aB^	0.67 ± 0.02 ^aA^

Mean values within each column (^a–d^) and row (^A–D^) with different superscript letters are significantly different at *p* < 0.05. L*, lightness; a*, redness; b*, yellowness; ΔE*, color differences.

## Data Availability

The raw data supporting the conclusions of this study will be made available by the authors upon request.

## Abbreviations

The following abbreviations are used in this manuscript:

ABTS	2,2′-azino-bis-(3-ethylbenzothiazoline-6-sulfonic acid)
ABTS+	ABTS cation radical
DPPH	2,2’-diphenyl-1-picryl-hydrazyl
DNS	3,5-dinitrosalicylic acid
FL	fructose–lysine
FLB	fructose–lysine–*β*-glucan
FRAP	ferric-reducing antioxidant power
GL	glucose–lysine
GLB	glucose–lysine–*β*-glucan
MRP	Maillard reaction product
PCA	principal component analysis
L*	lightness
a*	redness
b*	yellowness
ΔE*	color differences
WBN-EC	waxy-barley nurungji cooked using an electric rice cooker
WBN-PC	waxy-barley nurungji cooked using an electric rice pressure cooker
HBN-EC	hulled-barley nurungji cooked using an electric rice cooker
HBN-PC	hulled-barley nurungji cooked using an electric rice pressure cooker
BN0	control group
BN25	25% barley ratio
BN50	50% barley ratio
BN75	75% barley ratio
BN100	100% barley ratio
